# Identification of novel dysregulated circular RNAs in early‐stage breast cancer

**DOI:** 10.1111/jcmm.16324

**Published:** 2021-02-05

**Authors:** Arunagiri Kuha Deva Magendhra Rao, Vittal Rangan Arvinden, Deepa Ramasamy, Krishna Patel, Balaiah Meenakumari, Priya Ramanathan, Shirley Sundersingh, Velusami Sridevi, Thangarajan Rajkumar, Zdenko Herceg, Harsha Gowda, Samson Mani

**Affiliations:** ^1^ Department of Molecular Oncology Cancer Institute (WIA) Chennai India; ^2^ Institute of Bioinformatics ITPL Bangalore India; ^3^ Amrita School of Biotechnology Amrita Vishwa Vidyapeetham Kollam India; ^4^ Department of Oncopathology Cancer Institute (WIA) Chennai India; ^5^ Department of Surgical Oncology Cancer Institute (WIA) Chennai India; ^6^ Epigenetics Group International Agency for Research on Cancer (IARC) Lyon France; ^7^ Manipal Academy of Higher Education (MAHE) Manipal India; ^8^ QIMR Berghofer Royal Brisbane Hospital QLD Brisbane Qld Australia

**Keywords:** breast cancer, circRNA as microRNA precursor, circRNA‐microRNA interaction, circular RNA, non‐coding RNA

## Abstract

Breast cancer is a major cause of cancer‐related death in women worldwide. Non‐coding RNAs are a potential resource to be used as an early diagnostic biomarker for breast cancer. Circular RNAs are a recently identified group of non‐coding RNA with a significant role in disease development with potential utility in diagnosis/prognosis in cancer. In this study, we identified 26 differentially expressed circular RNAs associated with early‐stage breast cancer. RNA sequencing and two circRNA detection tools (find_circ and DCC) were used to understand the circRNA expression signature in breast cancer. We identified hsa_circ_0006743 (circJMJD1C) and hsa_circ_0002496 (circAPPBP1) to be significantly up‐regulated in early‐stage breast cancer tissues. Co‐expression analysis identified four pairs of circRNA‐miRNA (hsa_circ_0023990 : hsa‐miR‐548b‐3p, hsa_circ_0016601 : hsa_miR‐1246, hsa_circ_0001946 : hsa‐miR‐1299 and hsa_circ_0000117:hsa‐miR‐502‐5p) having potential interaction. The miRNA target prediction and network analysis revealed mRNA possibly regulated by circRNAs. We have thus identified circRNAs of diagnostic implications in breast cancer and also observed circRNA‐miRNA interaction which could be involved in breast cancer development.

## 1 INTRODUCTION

Breast cancer is the leading cause of cancer‐related deaths among women with 2.08 million new cases and 0.62 million deaths in 2018.^1^ Early detection can aid in better treatment outcomes. Non‐coding RNAs have been shown to be dysregulated in cancer with accumulating evidence for their potential utility in diagnosis and prognosis.^2^, ^3^, ^4^, ^5^ Circular RNAs (circRNA) are a large group of non‐coding RNAs that result from back splicing of linear RNA and are covalently closed circular single‐stranded RNA molecules lacking both 5′‐cap and 3′‐tail.^6^ Circular RNAs with ORF and IRES sequence can also code for peptides and therefore are a hybrid set of RNAs with both structural and functional importance.^7^ CircRNA can be exonic or contain a combination of exons, introns or untranslated regions of a linear RNA. The circRNAs harbouring UTR sequence and with intronic features may possess regulatory function. The deregulation of circular RNA expression can contribute to molecular events in carcinogenesis. In recent years, circRNAs are reported to be deregulated in hepatocellular carcinoma, oesophageal squamous cell carcinoma, colorectal cancer, bladder cancer and breast cancer.^8^ Interestingly, unique circRNA expression signature was observed in specific subtypes of breast cancer.^9^ Also, the deregulated circRNAs like circ_103110, circ_104689 and circ_104821 in breast cancer tissues have been implicated with diagnostic use in infiltrating ductal carcinoma.^10^ Circular RNAs are abundantly found in bodily fluids and also sorted into exosomes.^11^, ^12^ Hence, detectable circular RNAs specific to early‐stage breast cancer can be of immense importance in early diagnosis.

Understanding the functional role of circular RNAs in tumorigenesis and progression is still at its preliminary stage. The structure of circular RNA provides a longer half‐life, increasing the probability of interaction with other biomolecules.^13^ Most common role of competing endogenous RNAs is a well‐known function of circular RNA. Circular RNA *CDR1‐as* through its sponging activity was shown to hamper miR‐7‐mediated gene regulation. In addition, *CDR1‐as* can act as a buffer by sustained release of miR‐7 or as a reservoir when miR‐671 cleaves *CDR1‐as* using RISC assembly to mediate control of targets.^14^ Various circular RNAs have been shown to repress microRNA, altering its function in breast cancer. Hsa_circ_0001982, circABCB10 and circRAK3 were reported to be sponging miR‐143, miR‐1271 and miR‐3607 respectively leading to breast carcinogenesis and metastasis.^15^, ^16^, ^17^ Hence, the circRNAs mediated gene expression control by sponging microRNAs could be an early event which drives breast carcinogenesis. Whole transcriptome sequencing with an insight of circRNA, microRNA and mRNA expression will indicate the preliminary gene regulation orchestrated by circular RNAs.

In this study, we have attempted to identify circular RNAs differentially expressed in early‐stage breast cancer using RNA sequencing. The assessment of potential circular RNA‐microRNA interaction revealed multiple binding sites for microRNA, suggesting the sponging activity of circular RNA. In addition, we have also explored the function of circular RNAs harbouring sequences with the potential to yield mature microRNA. Overall, we have identified aberrantly expressed circular RNAs in early‐stage breast cancer, which may be used as stable diagnostic and prognostic biomarkers based on further evaluation.

## 2 MATERIALS AND METHODS

### 2.1 Study samples

Tissues were surgical specimens obtained from tumour bank at Cancer Institute (WIA), Chennai, India. Breast cancer cases of stage I‐IIA were selected for the study. Tumour tissue (N = 5) sections were histopathologically confirmed to contain more than 70% of tumour cells. Adjacent normal breast tissue from the same patient was used as matched normal samples (N = 5). From patients undergoing surgery for non‐malignant breast conditions, tissue samples distally away from the pathological indication within the breast were used as normal samples (N = 3, Table S3). All the surgical specimens were obtained after getting an informed consent from the participants. The study was conducted in accordance with the Declaration of Helsinki, and the protocol was approved by the Cancer Institute Ethical Committee, Cancer Institute (WIA).

### 2.2 Total RNA and small RNA sequencing

Total RNA isolation, ribosomal RNA depletion, and cDNA library preparation protocol have been detailed earlier, and sequencing data can be accessed at sequence read archive^18^ (Accession ID SRP156355). From the discovery set, microRNA was isolated using mirVana microRNA isolation kit (Thermo Fisher Scientific) and RNA integrity in each sample was evaluated using the Agilent Bioanalyzer RNA 6000 Nano assay (Agilent Technologies Inc.). SmallRNA libraries were constructed using TruSeq SmallRNA Sample Prep Kit (Illumina Technologies) following the manufacturer's instructions. Quality profile of library was evaluated using Bioanalyzer 2100 (Agilent Technologies Inc.) and 100 bp PE sequencing was carried out by HiSeq 2500 (Illumina, Technologies). The small RNA sequencing data have been submitted to sequence read archive (Reference ID PRJNA603126).

### 2.3 Identification of circular RNAs

FASTQ files were mapped against the human reference genome (hg38) using bowtie2. The unmapped reads from BAM files were used as input to find_circ algorithm. Briefly, the 20‐mer sequence from both ends of the unmapped reads was aligned against the anchor position within the spliced exons. BED files were filtered for circular RNAs with unambiguous breakpoints carrying characteristic GT/AG splice sites and a head‐to‐tail reverse alignment of the anchors. These circular RNAs obtained were filtered by five unique back‐spliced reads in at least one sample and ≥2 reads in a minimum of two out of other biological replicates of each group.

DCC algorithm was used to detect circular RNAs using the output from STAR read mapper and refine data with a series of inbuilt filters was also used.^19^ Reads were mapped to hg38 reference genome and ENSEMBL GrCh.38.37 GTF file using the STAR alignment tool to obtain information on chimeric splice junctions. The generated ‘chimeric.out.junction’ file contains chimerically aligned reads including circular RNA junction spanning reads. Circular RNAs identified by both tools were then annotated using circus R package using UCSC hg38 TxDB. The differential expression of circular RNAs detected by both find_circ and DCC was characterized using the DESeq2 R package. Circular RNAs with |fold change| ≥ 2 and p‐value ≤ 0.05 were considered to be differentially expressed between sample groups.

### 2.4 Validation of differentially expressed circular RNAs by Sanger sequencing and qRT‐PCR

The following primer sequences were used to amplify PCR fragments of three circular RNAs hsa_circ_0006743Fwd 5′‐GCCTGCATTGGTGTATGT‐3′; Rev 5′‐GCCCAGATTAAGTGGTATTCC‐3′, hsa_circ_0002496 Fwd 5′‐CTCATGAAGATTTGGCCTACT‐3′; Rev 5′‐TTACTACACAAGCAGTGCATT‐3′, hsa_circ_0023990 Fwd 5′‐TCTACATATGCAATAAGCTAGGATT‐3′; Rev 5′‐ TCGGAGGTAAGCCAAGAG‐3′ and amplifying Glyceraldehyde 3‐phosphate dehydrogenase gene fragment as internal reference using the primer sequences GAPDH Fwd 5′‐TGCACCACCAACTGCTTAGC‐3′; GAPDH Rev 5′‐ GGCATGGACTGTGGTCATGAG‐3′. Using GoTaq SYBR green master mix (Promega Corporation), circular RNA expression was validated using the above set of primers in Quantstudio^™^ 12K Flex (Applied Biosystems, Thermo Fisher Scientific) encompassing the junction site to ensure specificity. Genetic Analyzer 3500 DX (Applied Biosystems, Thermo Fisher Scientific) was used to confirm circular RNA sequences.

### 2.5 MicroRNA – circular RNA interaction prediction

Small RNA raw reads were trimmed and then aligned using STAR alignment tool following ENCODE microRNA‐SEQ pipeline. Differentially expressed miRNAs were identified using DESeq2. Spearman correlation analysis was performed to identify co‐expressed circular RNAs and microRNAs and a negative cut off value of Rho = −0.4 was considered. The mRNA targets of co‐expressed microRNAs were obtained from miRTarBase. Among these, only differentially expressed mRNAs from our data set were selected for visualizing the interaction using Cytoscape. To identify the deregulated pathways, Reactome was used with targets of negatively correlated microRNAs as input. For circular RNAs acting as microRNA precursors, the mature sequences of circular RNA were searched for stem‐loop microRNA sequence using BLAST tool in miRBase.

## 3 RESULTS

### 3.1 Expression signature of circular RNAs in breast cancer

**FIGURE 1:**
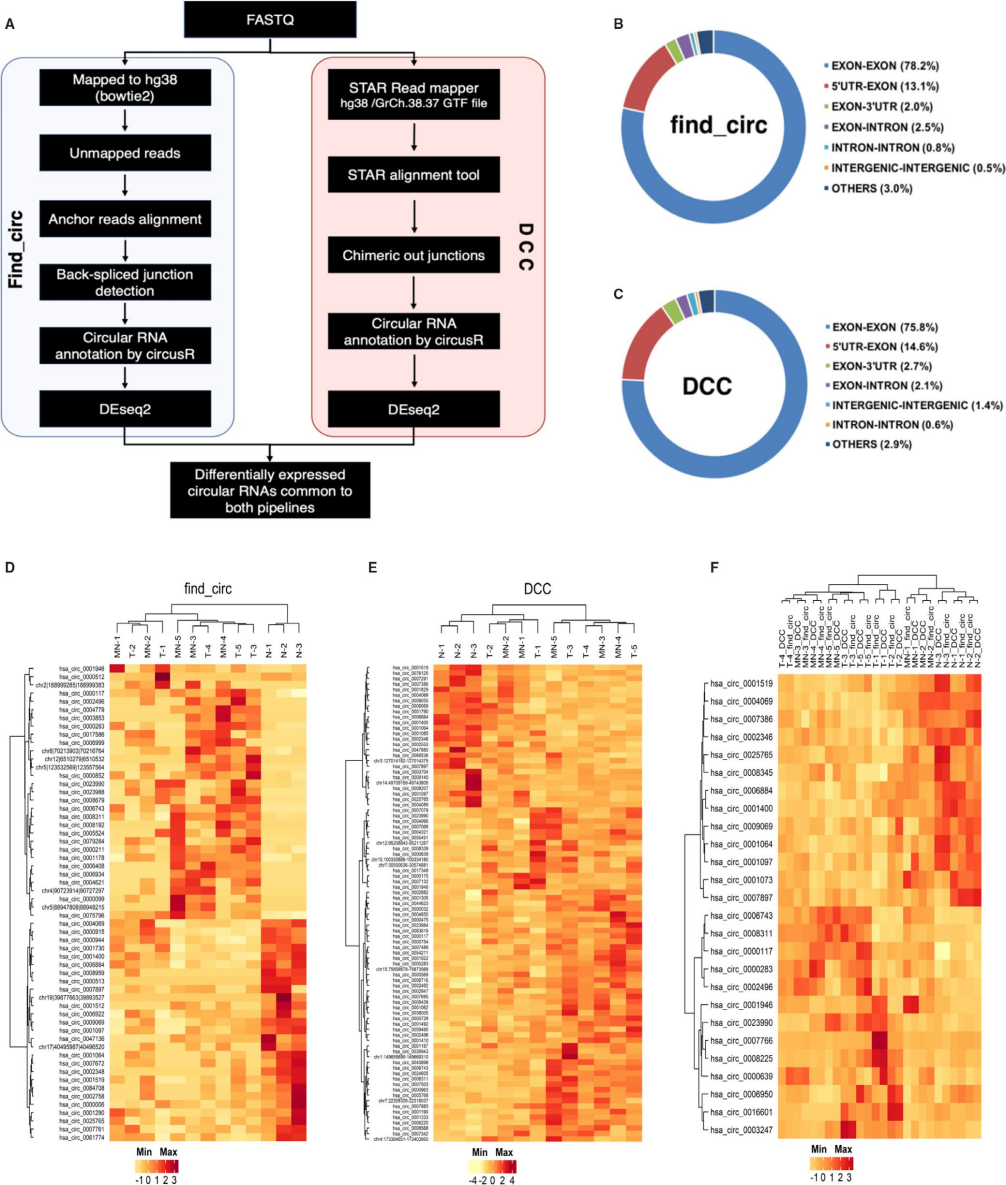
Identification of circular RNAs differentially expressed in breast cancer, matched normal and apparent normal samples. A, Schematic pipeline employed for circular RNA identification. B, Genomic features of circRNAs identified by find_circ. C, Genomic features of circRNAs identified by DCC. D, Heatmap showing the 58 deregulated circRNAs in tumour samples compared to matched normal and apparent normal samples identified using find_circ. E, Heatmap showing the 87 deregulated circRNAs in tumour samples compared to matched normal and apparent normal samples identified using DCC. F, Heatmap of 26 circRNAs dysregulated in tumour samples compared to matched normal and apparent normal samples common to both find_circ and DCC

RNA sequencing of breast tissues resulted in approximately 89 million reads which were filtered for unmapped reads using bowtie2. The unmapped reads were used for identification of circular RNAs using two different algorithms, find_circ and DCC. Find_Circ tool was used to identify circular RNAs (Figure 1A). About 1769 (find_circ) and 1413 (DCC) circRNAs were found to carry unique back‐spliced reads in at least three samples (Table 1). The genomic features of identified circular RNAs show that exonic circular RNAs are the most abundant class accounting for 78% obtained from find_circ and 75.8% from DCC. Circular RNAs consisting of 5′UTR‐exon (13.1% and 14.6%) and exon‐3′UTR sequence (2% and 2.7%) also contributed to the number of identified circular RNAs through find_circ and DCC respectively (Figure 1B,C). Circular RNA arising from exon‐intron and intergenic regions and others (5′UTR‐3′UTR, UTR‐tx, exon‐tx., etc) were ranging from 2.9% to 0.5%. The results show that the majority of circular RNAs carry exon‐exon sequence arising due to mRNA splicing and circularization.

**TABLE 1:** Total number of identified circular RNAs by find_circ and DCC

Samples	Find_circ		DCC‐identified circular RNAs	Number of circular RNAs in common
	Number of circular RNAs	Filtered circular RNAs		
T‐1	7698	560	1194	421
T‐2	10 309	1055	1392	700
T‐3	12 415	1396	1395	828
T‐4	19 041	1632	1412	921
T‐5	11 436	1373	1403	840
MN‐1	6475	695	1307	510
MN‐2	12 717	1269	1405	813
MN‐3	10 987	1323	1392	821
MN‐4	12 857	1239	1350	767
MN‐5	6265	918	1303	611
N‐1	21 214	1604	1409	926
N‐2	15 729	1385	1394	869
N‐3	7000	885	1179	643

Abbreviations: T, tumour; MN, matched normal; N, apparent normal.

### 3.2 Differentially expressed circular RNAs in breast tumours

A total of 1516 and 1270 circular RNAs were annotated using CircBase. Hierarchical clustering shows the grouping of samples distinctively into tumour‐matched normal and apparent normal (Figure 1D,E and F). Hsa_circ_0001064, Hsa_circ_0001073, Hsa_circ_0001097 and Hsa_circ_0001400 were expressed at relatively low levels in both tumour and matched normal tissues compared to apparent normal tissues. Two circular RNAs, hsa_circ_7386 (circCRIM1) and hsa_circ_8345 (circPTK2) was down regulated in tumour relative to both matched normal and apparent normal samples. DEseq2 analysis on circular RNA identified by find_circ showed 58 differentially regulated circular RNAs (30 up‐regulated and 28 down‐regulated), whereas 87 deregulated circular RNAs consisting of 61 up‐regulated and 26 down‐regulated candidates resulted from DCC‐identified circular RNAs. Several common deregulated circular RNAs were found when tumour samples were compared to matched normal and normal separately (Tables S1 and S2). In total 26 circular RNAs (13 up‐regulated and 13 down‐regulated) were found to be differentially regulated in both find_circ and DCC based analysis (Table 2).

**TABLE 2:** List of differentially regulated circular RNAs in early‐stage breast cancer

Circular RNA	MN‐T vs N (log_2_FC)		T vs MN (log_2_FC)		T vs N (log_2_FC)		Parental gene
	DCC	find_circ	DCC	find_circ	DCC	find_circ	
hsa_circ_0000117	1.13	1.08	‐	‐	1.11	1.05	MAN1A2
hsa_circ_0000283	1.65	2.63	‐	‐	‐	‐	CSTF3
hsa_circ_0000639	‐	‐	‐	‐	2.05	2.61	ETFA
hsa_circ_0001064	‐1.17	‐2.68	‐	‐	‐	‐	EPB41L5
hsa_circ_0001073	‐	‐	‐	‐	‐1.56	‐1.32	ACVR2A
hsa_circ_0001097	‐1.37	‐1.51	‐	‐	‐	‐	PIKFYVE
hsa_circ_0001400	‐1.25	‐1.35	‐	‐	‐1.20	‐1.36	RELL1
hsa_circ_0001519	‐1.24	‐1.45	‐	‐	‐1.25	‐1.34	MAN2A1
hsa_circ_0001946	2.10	1.58	‐	‐	2.00	1.47	AL078639.1
hsa_circ_0002346	‐	‐	‐	‐	‐2.10	‐1.85	CRIM1
hsa_circ_0002496	2.28	4.02	‐	‐	2.42	4.10	APPBP1
hsa_circ_0003247	‐	‐	‐	‐	1.19	1.09	ASH1L
hsa_circ_0004069	‐1.30	‐1.43	‐	‐	‐1 0.72	‐2.01	SLC37A3
hsa_circ_0006743	2.18	4.77	‐	‐	‐	‐	JMJD1C
hsa_circ_0006884	‐1.17	‐1.5	‐	‐	‐1.12	‐1.45	TIMMDC1
hsa_circ_0006950	‐	‐	1.37	1.97	‐	‐	TRPS1
hsa_circ_0007386	‐	‐	‐	‐	‐1.71	‐1.65	CRIM1
hsa_circ_0007766	‐	‐	3.14	3.03	‐	‐	ERBB2
hsa_circ_0007897	‐1.17	‐1.98	‐	‐	‐	‐	BOC
hsa_circ_0008225	‐	‐	2.19	2.49	1.96	2.01	ZMYND11
hsa_circ_0008311	1.13	1.25	‐	‐	‐	‐	STAM
hsa_circ_0008345	‐	‐	‐	‐	‐1.29	‐1.64	PTK2
hsa_circ_0009069	‐1.06	‐1.61	‐	‐	‐	‐	PHF8
hsa_circ_0016601	‐	‐	3.58	3.58	‐	‐	DNAH14
hsa_circ_0023990	1.72	2.43	‐	‐	1.99	2.85	NOX4
hsa_circ_0025765	‐1.59	‐1.73	‐	‐	‐2.01	‐1.96	TMTC1

Abbreviations: T, tumour; MN, matched normal; N, normal; Log2FC, p < 0.05

Based on normalized read counts from both pipelines, the expression status was plotted to specifically identify candidate circular RNAs differentially expressed in early‐stage breast cancer. Among the up‐regulated circular RNAs, hsa_circ_0001946 (CDR1as) and hsa_circ_0007766 (circERBB2) showed a high expression in tumour tissues and matched normal tissue when compared with apparent normal tissues. Expression levels were high for hsa_circ_0006743 (circJMJD1C), hsa_circ_0002496 (circAPPBP2) and hsa_circ_0023990 (circNOX4) in both tumour and matched normal tissue. Hsa_circ_0008225 (circZMYND11) and hsa_circ_0016601 (circDNAH14) were found to be unregulated in tumour tissues compared to matched normal and normal tissue.

### 3.3 Validation of early‐stage breast cancer associated circular RNAs

**FIGURE 2:**
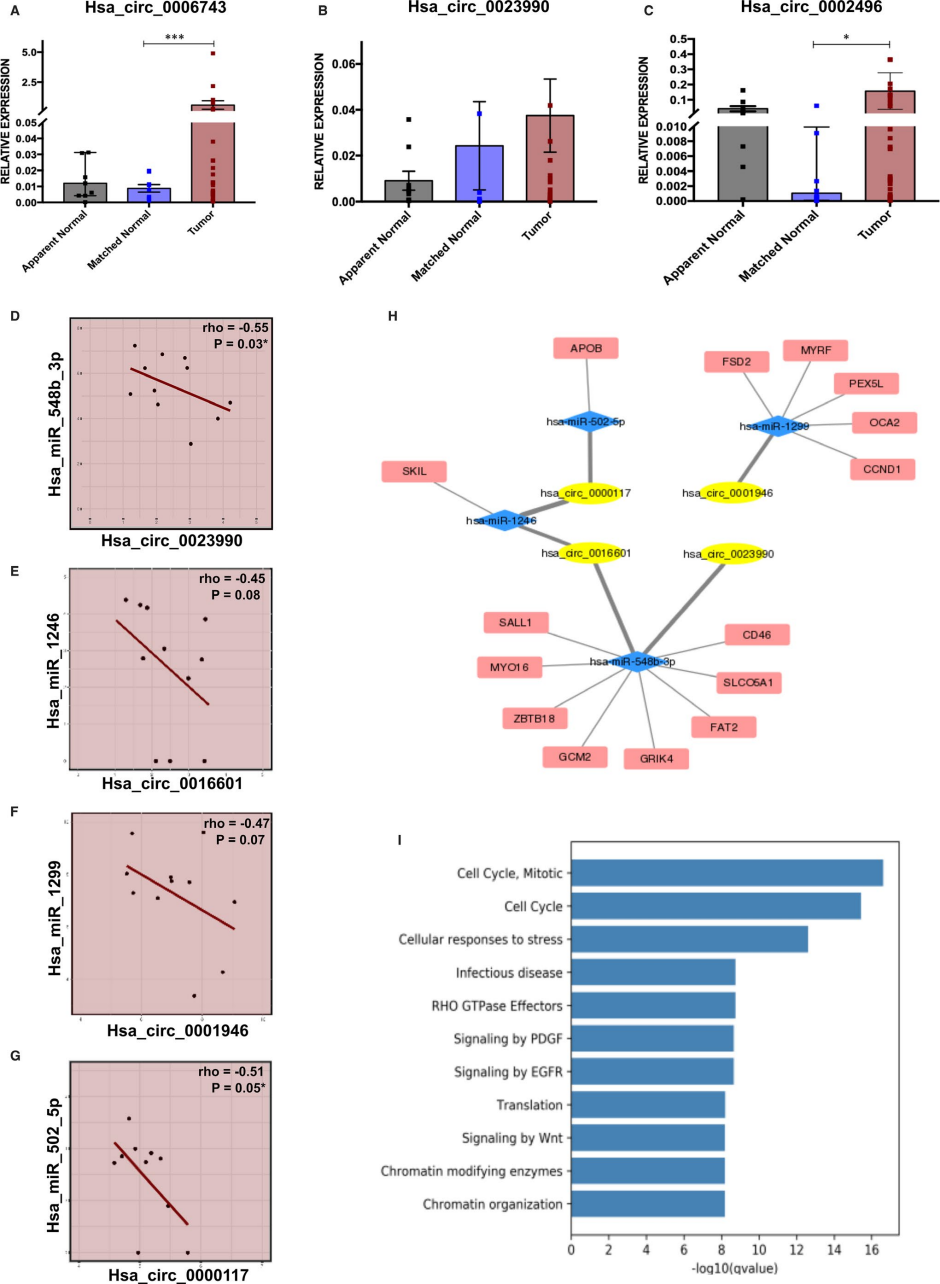
Validation and integrated analysis of differentially expressed circRNAs in early‐stage breast cancer. Validation of differential expression of circRNAs by qRT‐PCR analysis A, Hsa_circ_0006743; B, Hsa_circ_0023990; C, Hsa_circ_0002496 [T‐test for unpaired samples was applied and p value ‐ * (≤0.05), ** (≤0.01) & *** (≤0.001) were considered statistically significant]. Spearman correlation analysis of circular RNA and microRNA expression showing a negative association (Rho ≤ −0.4) between D, Hsa_circ_0023990 and hsa_miR_548_3p; E, Hsa_circ_0016601 and hsa_miR_1246; F, Hsa_circ_001946 and hsa_miR_1299; G, Hsa_circ_0000117 and hsa_miR_502_5p; H, Network analysis of circular RNA, microRNA and target mRNA showing an indirect regulation of gene expression mediated by circular RNA; I, Reactome pathway analysis showing cancer‐associated pathways deregulated by circRNAs

To confirm the identified circular RNA, the junction sequence was verified from the RNA sequencing data. Partial sequence was compared with the sequence obtained from circRNAdb and region of circularization or junction sequence was confirmed by Sanger sequencing (Figure S1). Three DEcircRNAs were validated by qRT‐PCR in a set of tumour (N = 32), matched normal (N = 9) and apparent normal (N = 9) samples. Hsa_circ_0006743 was found to be up‐regulated in tumour samples (Relative mean expression, SEM, p value) (0.597, ±0.349) compared to matched normal samples (0.009, ±0.002, p = 0.006) and apparent normal samples (0.022, ±0.01, p = 0.058) (Figure 2A). Hsa_circ_0023990 was also up‐regulated in tumour samples (0.037, ±0.015) compared to matched normal samples (0.024, ±0.019, p = ns) and apparent normal samples (0.009, ±0.004, p = .059) but was not statistically significant (Figure 2B). Similarly, Hsa_circ_0002496 was overexpressed in tumour samples (0.158, ±0.058) relative to matched normal samples (0.008, ±0.006, p = 0.046) and apparent normal samples (0.041, ±0.017, p = ns) (Figure 2C). The results confirm that hsa_circ_0006743 and hsa_circ_0002496 are up‐regulated in early‐stage breast cancer.

### 3.4 MicroRNA sponging by circular RNA

Small RNA sequencing was performed to correlate circular RNA and microRNA expression. Small RNA sequencing was performed on the same set of samples, which resulted in 45 million reads. A total of 659 microRNA were found to be negatively correlated with 26 DEcircRNAs (Spearman correlation coefficient, rho ≤ −0.4). Four circRNA:microRNA pairs (hsa_circ_0023990:hsa‐miR‐548b‐3p,hsa_circ_0016601:hsa_miR‐1246, hsa_circ_0001946:hsa‐miR‐1299 and hsa_circ_0000117:hsa‐miR‐502‐5p) were found significant in negative correlation (rho = −0.45 to −0.55) (Figure 2D,E,F and G). Also, complete sequence of DEcircRNAs hsa_circ_0023990, hsa_circ_0016601, hsa_circ_0001946 and hsa_circ_0000117 were confirmed to carry binding sites for the four microRNAs, hsa‐miR‐548b‐3p, hsa‐miR‐1246, hsa‐miR‐1299 and hsa‐miR‐502‐5p, respectively. Using Cytoscape, a comprehensive association between mRNA targets, microRNAs and circRNAs was developed to observe the underlying inter‐relationship. The overall network interaction consists of 26 circRNA nodes, 659 microRNA nodes, 889 mRNA nodes and 8041 edges connecting them (Figure S2). A more refined interaction between the negatively correlated circRNAs and microRNAs consisted of four circRNAs and four microRNAs with 15 downstream target mRNAs (Figure 2H). Pathway Enrichment Analysis using *Reactome* database highlighted cell cycle processes, response to stress, chromatin modifications and cellular EGFR, PDGF and WNT signalling pathways as major biological processes being altered (Figure 2I). These findings elucidate the differential expression of circRNA can potentially modulate breast cancer associated pathways through microRNA sponging at an early stage of tumour development.

### 3.5 Circular RNA can act as precursors for microRNA

The secondary structure of circular RNAs showed several stem loop structures resembling pre‐microRNA structure. The mature circular RNA sequences were checked for carrying pre‐microRNA that can generate microRNA. Of the circular RNAs identified, hsa_circ_0001519 was found to contain precursor sequences for five microRNA while hsa_circ_0007766 contained precursor sequences for four microRNA (Table 3). Five circular RNAs, namely hsa_circ_0002346, hsa_circ_0001064, hsa_circ_0002496, hsa_circ_0000639 and hsa_circ_0001073, were predicted to yield three microRNA each. Hsa_circ_0007766 was predicted to give rise to miR‐370, commonly up‐regulated microRNA in breast cancer. Besides, miR‐141 and miR‐372 were predicted to be processed from circ_0008225 and circ_0002496 respectively had been previously reported to possess tumour suppressive property.

**TABLE 3:** List of circular RNAs predicted to be a precursor for microRNAs

Circular RNA	Number of microRNAs originated	MicroRNA
hsa_circ_0001519	5	hsa‐miR‐5195; hsa‐miR‐3913‐1; hsa‐miR‐3913‐2; hsa‐miR‐6864; hsa‐miR‐379
hsa_circ_0007766	3	hsa‐miR‐6865; hsa‐miR‐3155a; hsa‐miR‐370
hsa_circ_0002346	3	hsa‐miR‐1263; hsa‐miR‐12118; hsa‐miR‐7114
hsa_circ_0002496	3	hsa‐miR‐372; hsa‐miR‐450b; hsa‐miR‐450a‐2
hsa_circ_0001064	3	hsa‐miR‐3612; hsa‐miR‐4447; hsa‐miR‐9986
hsa_circ_0000639	3	hsa‐miR‐2681; hsa‐miR‐548k; hsa‐miR‐4299
hsa_circ_0001073	3	hsa‐miR‐6129; hsa‐miR‐449a; hsa‐miR‐563
hsa_circ_0008311	2	hsa‐miR‐6504; hsa‐miR‐517a
hsa_circ_0001097	2	hsa‐miR‐211; hsa‐miR‐548ay
hsa_circ_0008225	2	hsa‐miR‐141; hsa‐miR‐19a
hsa_circ_0007386	2	hsa‐miR‐1263; hsa‐miR‐7114
hsa_circ_0006884	2	hsa‐miR‐3152; hsa‐miR‐548am
hsa_circ_0007897	2	hsa‐miR‐1266; hsa‐miR‐3085

## 4 DISCUSSION

Circular RNAs are emerging non‐coding RNAs recognized with immense potential in controlling regulatory. Several studies have established differential expression of circular RNAs in tumour tissues compared to normal tissue in wide range of cancer types.^20^ Circular RNAs were also detectable in circulation especially enriched in exosomes indicating their significance as non‐invasive tumour biomarkers.^21^ Recent findings on circular RNA/microRNA axis of gene expression regulation demonstrate a higher degree of complexity. Therefore, interrogating circular RNAs can uncover multiple fundamental mechanisms in cancer. Identification of circular RNAs is challenging with the current tools available. Most tools identify circular RNAs based on back‐splice junction with minor variations.^22^

In our study, we combined the power of RNA sequencing that enables a comprehensive analysis of RNA expression across the transcriptome, with two different pipelines to detect circular RNAs in a set of tumour, matched normal and apparent normal samples. Find_circ utilizes the unmapped reads from bowtie2 to identify back‐spliced junctions while DCC detects circular RNA through splice aware aligner STAR. The number of deregulated circular RNAs varied between find_circ and DCC but 944 common circular RNAs were found among a total of 1769 (find_circ) and 1413 (DCC) predicted circular RNAs. It was interesting to observe that the genomic landscape of most circular RNAs was exonic followed by 5′UTR‐exon and exon‐3′UTR, implicating major contribution from mRNA splicing. The circular RNA transcripts with intron and UTR sequence can sequester microRNAs especially the exonic‐3′UTR circular RNAs which may harbour multiple microRNA response elements. Up‐regulation of such circular RNAs will compete for microRNA affecting target mRNA levels. Our results indicate high expression of hsa_circ_0006743, hsa_circ_0002496, and hsa_circ_0023990 in breast cancer tissues. To our knowledge this is the first study reporting on these circular RNAs to be deregulated in breast cancer, although hsa_circ_0023990 were found to be up‐regulated in thyroid carcinomas and colorectal cancers.^23^, ^24^ Further, hsa_circ_0000639 and hsa_circ_0025765 were overexpressed in both breast tumour tissues and matched normal tissues, while hsa_circ_0007766 (circERBB2) and hsa_circ_0016601 (circDNAH14) were up‐regulated exclusively in tumour tissues. CircERBB2 and circDNAH14 have been previously associated with gastric cancer and endometrial cancer respectively.^25^, ^26^ Further, the expression of hormone receptors – ER, PR and HER2/nue were correlated with circRNA expression and no significant association was found (Figure S3). To understand the role of circRNA in breast cancer prognosis, survival analysis was carried out. The survival analysis based on circRNA expression showed no association (Figure S4). Since all the samples tested were of stage I, an early clinical intervention possible resulted in better survival rates. Nevertheless, analysis using advanced stage or metastatic breast cancer cases may provide a definite prognostic role of the differentially expressed circRNA.

Circular RNA‐microRNA interaction has proved to be a potential mechanism of gene regulation that plays a significant role in breast cancer progression. The sponging of miR‐1271 and miR‐143 by circ‐ABCB10 and hsa_circ_0001982 has been demonstrated earlier with breast cancer.^15^, ^16^ In our study, we used both small RNA sequencing and total RNA sequencing to obtain a comprehensive expression profile of microRNAs, circular RNAs and mRNAs. We observed a group of co‐expressed miRNAs that were negatively correlated with the differentially expressed circular RNAs. Our co‐expression analysis represents the plausible role of circular RNA in regulating mRNA expression through sponging or decoying microRNAs. CCND1, SKIL and CD46 primarily involved in early breast cancer progression and immune evasion were predicted to be dysregulated by circRNA‐miRNA interaction. Our results showed that hsa_circ_0001946, also known as CDR1*as* or ciRS‐7, is differentially regulated in early‐stage breast tumours. The oncogenic potential of hsa_circ_0001946 is known to occur by sponging of miR‐7 in different tumour types.^27^ In addition, CDR1*as* also sponges other microRNAs like miR‐135a, miR‐876‐5p and miR‐671‐5p that have cancer implications.^28^, ^29^, ^30^ In our study, we found CDR1*as* or hsa_circ_0001946 to be negatively correlated with miR‐26a, miR‐342, miR‐30b, miR‐214, miR‐125b, miR‐196a, miR‐92b and miR‐19a. These miRNAs are described to be associated with breast cancer development indicating possible new hsa_circ_0001946‐miRs axes.^31^, ^32^, ^33^, ^34^, ^35^, ^36^, ^37^ Another up‐regulated circRNA, hsa_circ_0002496 negatively correlated with miR‐125B1, miR‐204 and miR‐10B implicating potential sponging and since these microRNAs being associated with breast cancer is a significant observation of the study.^35^, ^38^, ^39^ Nevertheless, it is warranted to evaluate the regulatory relationship between circular RNA and microRNA experimentally.

In summary, we identified 26 aberrantly expressed circRNAs in early‐stage breast cancer. Three circRNAs – hsa_circ_0006743, hsa_circ_0002496 and hsa_circ_0023990 – were confirmed to be specifically up‐regulated in early‐stage breast cancer. Integrated co‐expression analysis revealed the sponging role of circRNAs in breast cancer. Interestingly, some circRNAs were also found to harbour pre‐microRNA sequence, possessing a possibility to act as precursors for microRNA. Breast cancer‐associated novel circRNAs were reported in this study and can be explored for diagnostic and prognostic potential.

## CONFLICT OF INTEREST

The authors declare no conflict of interest.

## AUTHOR CONTRIBUTIONS

Arunagiri Kuha Deva Magendhra Rao: Formal analysis (lead); Methodology (lead); Validation (lead); Visualization (lead); Writing‐original draft (lead). Arvinden Vittal Rangan: Formal analysis (equal); Methodology (equal); Visualization (equal). Deepa Ramasamy: Formal analysis (supporting). Krishna Patel: Formal analysis (supporting). Meenakumari Balaiah : Methodology (supporting). Priya Ramanathan: Writing‐review & editing (supporting). Shirley Sundersingh: Resources (lead). Sridevi Velusami : Resources (lead). Rajkumar Thangarajan : Writing‐review & editing (supporting). Zdenko Herceg: Writing‐review & editing (supporting). Harsha Gowda: Formal analysis (supporting). Samson Mani: Conceptualization (lead); Funding acquisition (lead); Investigation (lead); Supervision (lead); Writing‐review & editing (lead).

## DISCLAIMER

Where authors are identified as personnel of the International Agency for Research on Cancer / World Health Organization, the authors alone are responsible for the views expressed in this article and they do not necessarily represent the decisions, policy or views of the International. Agency for Research on Cancer / World Health Organization.

## Supporting information

Figures S1‐S4

Table S1

Table S2

Table S3

## Data Availability

The data that support the findings of this study are available from the corresponding author upon reasonable request.
