# Supplementary material for: Identification of novel dysregulated circular RNAs in early‐stage breast cancer
Source: J Cell Mol Med. 2021 Feb 5;25(8):3912–21. doi: 10.1111/jcmm.16324 (PMC8051735; doi:10.1111/jcmm.16324)
Supplement: Supplementary file 1 — Figures S1‐S4 [file JCMM-25-3912-s001.pdf]

Rao *et al*., Identification of novel dysregulated circular RNAs in early-stage breast cancer

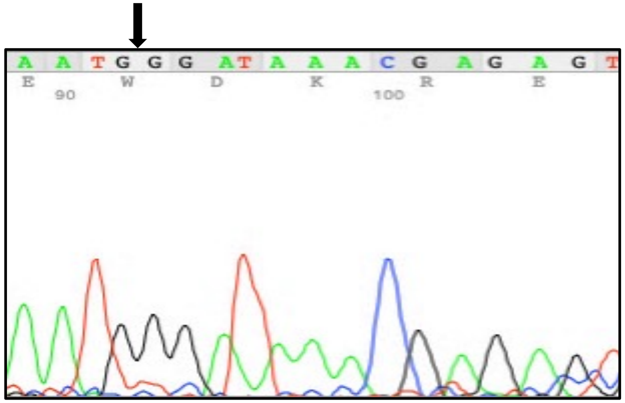

Hsa_circ_0006743

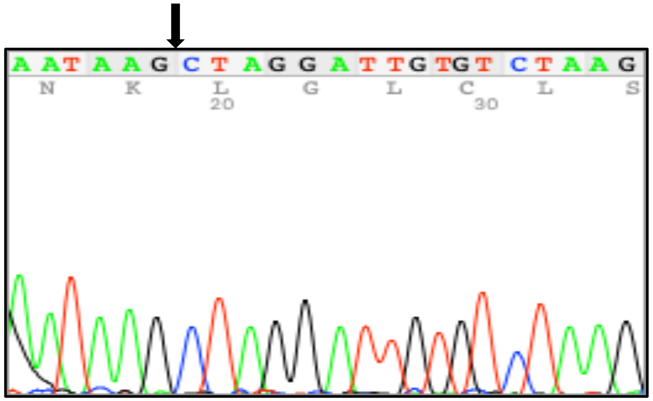

Hsa_circ_0023990

Figure S1. Sanger sequencing results of hsa_circ_0006743 and hsa_circ_0023990 showing the splice junction site (Arrow indicates junction site)

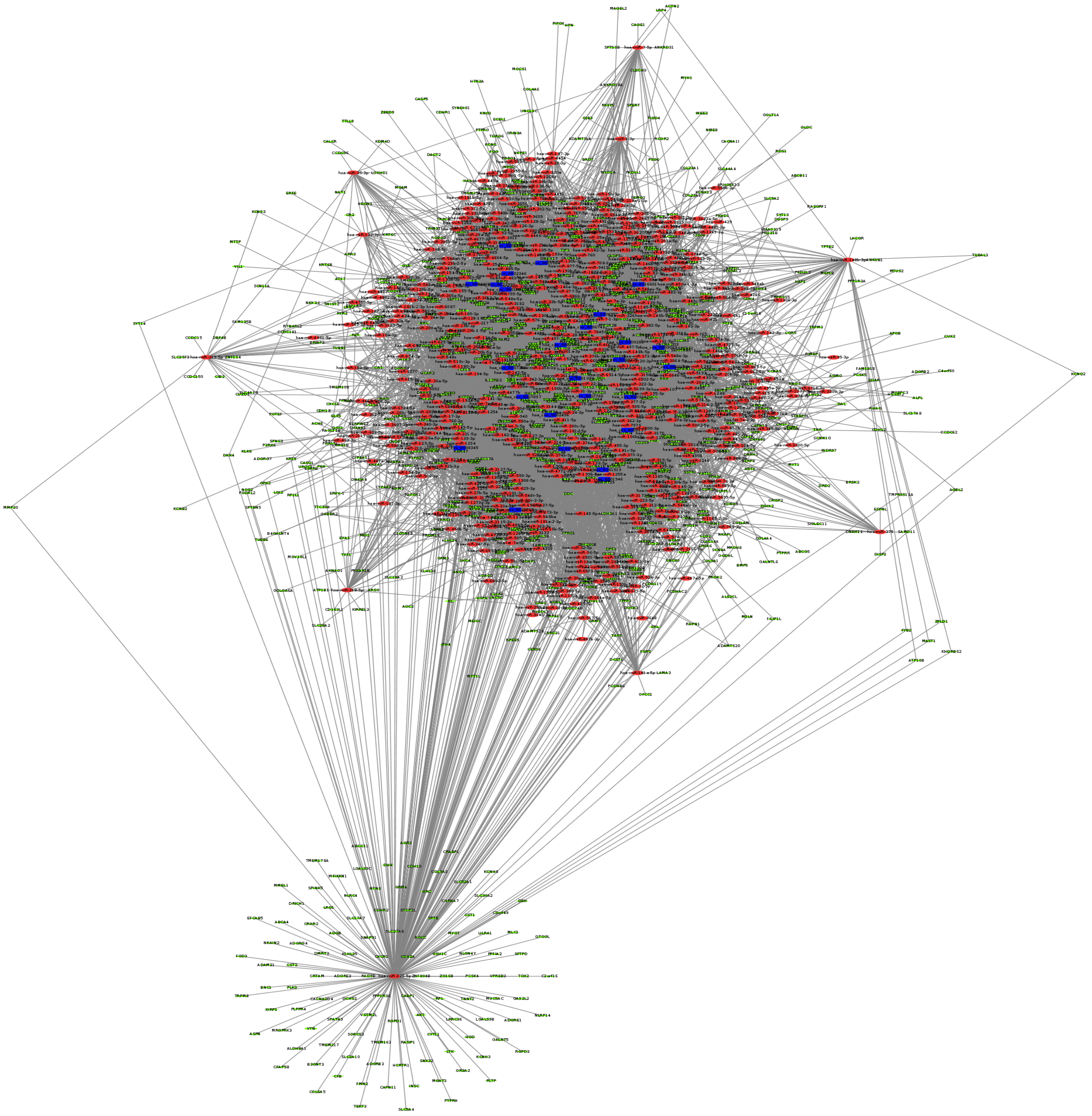

Figure S2. Cytoscape network of complete set of negatively correlated circular RNAs and microRNAs (Blue nodes – ciruclar RNAs, Red nodes – microRNAs and Green nodes – target mRNAs)

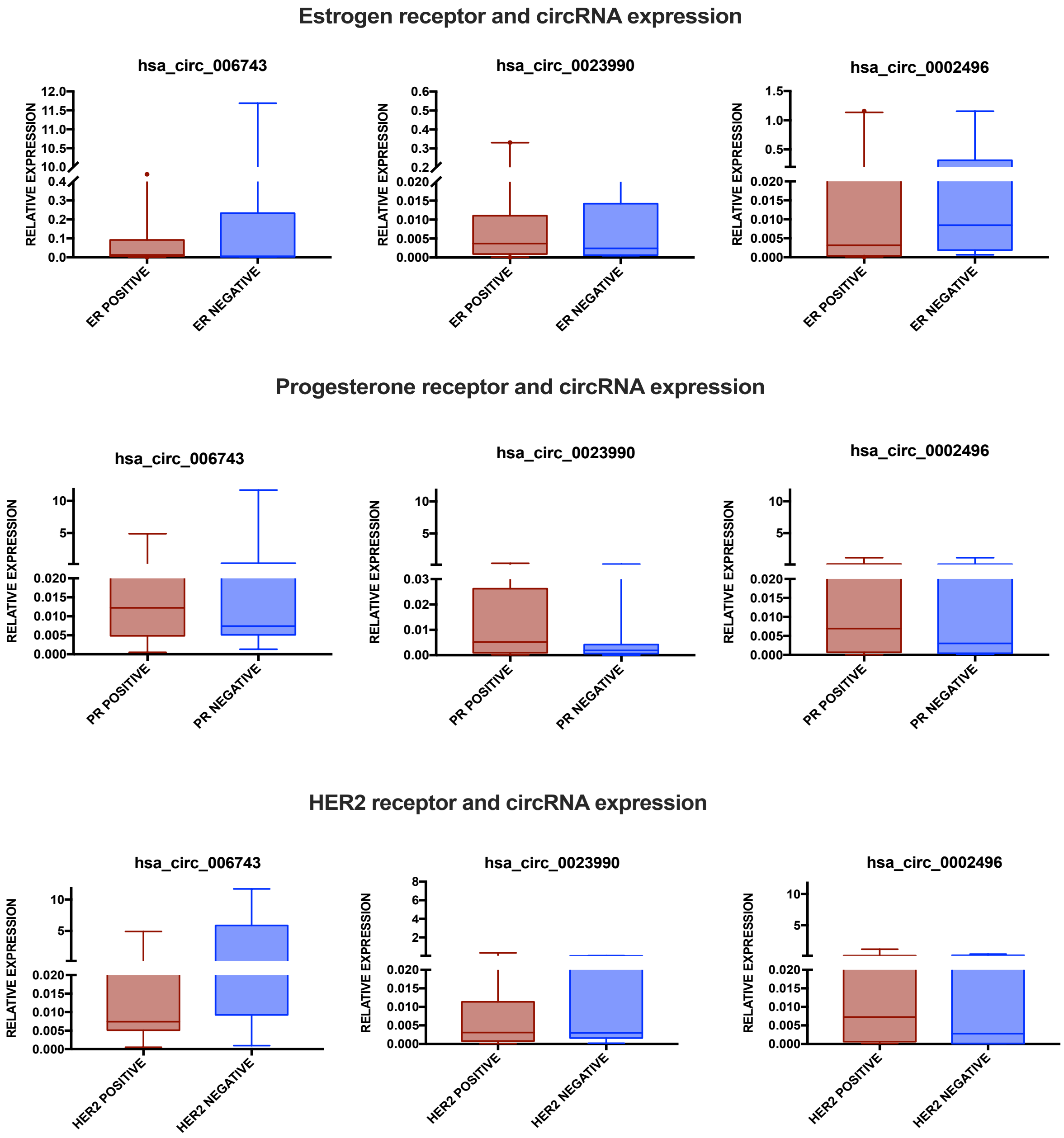

Figure S3. Estrogen Receptor, Progesterone Receptor and Human Epidermal Growth Factor Receptor 2 expression and circRNA expression analysis (P value for unpaired t test were greater than 0.05)

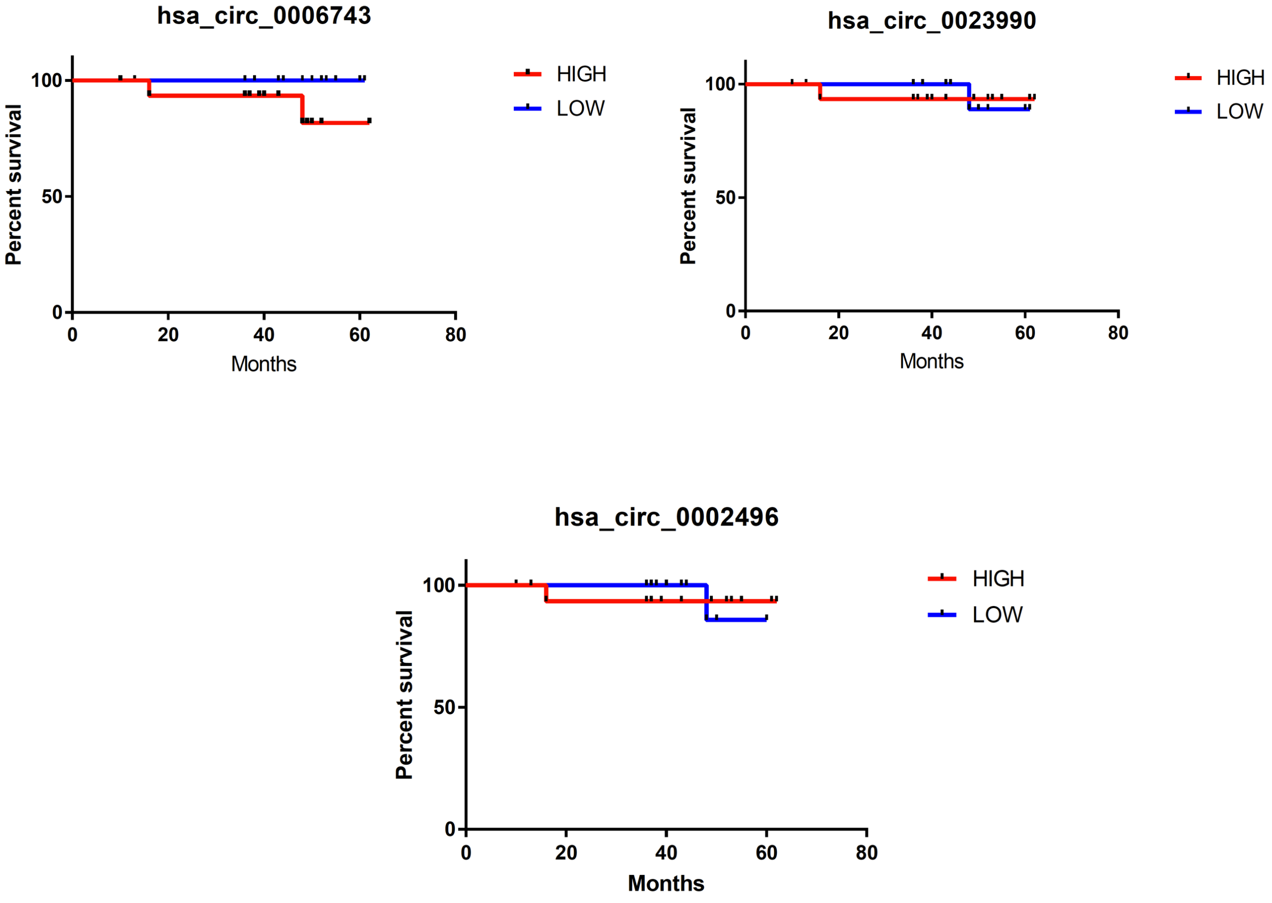

Figure S4. Kaplan-Meier Plot showing overall survival in association with circRNA expression
